# Effect of feeding chayote (*Sechium edule*) meal on growth performance and nutrient utilization in indigenous pig (Zovawk) of Mizoram

**DOI:** 10.14202/vetworld.2015.918-923

**Published:** 2015-07-26

**Authors:** James Lalthansanga, A. K. Samanta

**Affiliations:** Department of Animal Nutrition, College of Veterinary Sciences & Animal Husbandry, Central Agricultural University, Selesih, Aizawl - 796 001, Mizoram, India

**Keywords:** chayote, indigenous pigs, growth performance, nutrient digestibility

## Abstract

**Aim:**

This study was planned to investigate the effect of feeding different levels of chayote (*Sechium edule*) meal by replacing standard concentrate mixture (CM) on the growth parameters such as feed intake, body weight gain, average daily gain (ADG) and feed conversion ratio (FCR), and nutrient utilization in indigenous pig of Mizoram.

**Materials and Methods:**

Twenty-four growing indigenous pigs (Zovawk) were used to study the effect of feeding chayote (*Sechium edule*) meal (fruits and leaves at the ratio 4:1) on growth performance and nutrient utilization. They were allocated randomly into 4 treatment groups (G_1_, G_2_, G_3,_ and G_4_). Chayote meal was used to replace standard CM (pig grower ration) at 0% (G_1_), 20% (G_2_), 30% (G_3_), and 40% (G_4_).

**Results:**

During the feeding trial of 90 days, it was found that the dry matter (DM) intake decreased as the level of chayote meal increased. For G_1_, G_2_, G_3_, and G_4_, the ADG (kg) was 0.24±0.04, 0.23±0.03, 0.18±0.02, and 0.18±0.02, respectively, and the feed conversion efficiency was 5.42±0.44, 4.93±0.17, 5.38±0.05, and 5.74±0.53, respectively. However, there was no significant difference (p>0.05) among the different treatment groups in respect to ADG and FCR. At the end of the feeding trial, digestibility trial was conducted to study the effect of feeding chayote meal in the digestibility of the different nutrients by the experimental animals. From the digestibility trial, it was revealed that the digestibility coefficient of DM, crude protein, and crude fiber were also similar (p>0.05), although the ether extract digestibility in G_1_ was significantly low (p<0.01) as compared to G_2_, G_3_, and G_4_.

**Conclusion:**

Chayote meal could safely replace the standard grower ration up to 40% in the diet of growing local pigs without causing any adverse effects on growth and nutrient utilization.

## Introduction

Mizoram, one of the states of the North-Eastern hilly region of India is situated between the 20.58° and 23.35° north latitude and 92.15° and 93.29° east longitude. Agriculture has been one of the main occupations in Mizoram in which animal husbandry occupies a potential source of rural economy. Among the livestock species, pig is by far the most populous and popular livestock in Mizoram and shows the highest percentage of growth in its population.

Feed cost accounts to 70-85% of total recurring expenditure [1] and therefore an important aspect in pig husbandry is to minimize the cost of feeding by the utilization of unconventional feedstuffs such as sweet potato, chayote, tapioca, etc. which are available locally. The expenditure of feeding pigs with unconventional feed varies considerably with productive traits of the pig and marketing potential of locally available feedstuffs [2].

Chayote or Squash, locally called “Iskut” in Mizoram has been cultivated extensively and utilized for human consumption as well as pig feed. The state probably has the largest area under squash cultivation in India, though it is also grown in other states of the North East. The area under cultivation of chayote in Mizoram has recorded steady increase with a production of 24,455 million tons in 2006-2007 [3]. During the peak season, plenty of squash fruits are available in the market, the price of which even goes down to Rs. 2 per kilogram [4]. The chayote plant yields shoots which are used as vegetable greens, vines as an ornament for fences or as animal fodder, and edible subterranean storage roots [5].

Scientific information on the use of chayote fruits and leaves in pig ration are not available. Keeping in view the above mentioned facts, the research was carried out with the objective of studying the growth and nutrient utilization of Zovawk pigs fed on different levels of chayote (*Sechium edule*) meal (fruits and leaves) in the diet.

## Materials and Methods

### Ethical approval

The research was carried out as per the guidelines in force at the time of carrying out the experiment and approval of Institutional Animal Ethics Committee.

### Experimental animals and design

The experimental animals used were an indigenous breed of pigs found in Mizoram, locally called Zovawk. Zovawk is small type local pigs. Males are bigger than females and can attain full growth in 3 years and weigh as much as 60-70 kg, while the females may be as much as 50-55 kg in live weight. The body is stout with a short neck. The abdomen is large and can sometimes touch the ground, while standing which conform an arch back. The rump region is smooth and the tail is a long bearing switch with long hairs. 24 indigenous breed of pigs of average body weights were allocated randomly into 4 treatment groups (G_1_, G_2_, G_3_, and G_4_) and housed in the well-ventilated animal shed. A provision for feeding individually twice a day was arranged. Fresh drinking water was provided at all times. The duration of the feeding trial was 90 days.

### Dietary treatment

A Grower–Finisher ration was prepared [6]. This mixture was considered as a Standard Concentrate mixture (CM). The ingredient composition of the standard CM was yellow maize 55%, wheat bran 20%, groundnut cake 16%, fish meal 7%, mineral mixture 1.5%, and common salt 0.5%. Three types of rations that are iso-nitrogenous to the standard CM_S_ were prepared for feeding to the three different groups (G_2_, G_3_, and G_4_) of animals. Chayote meal was used to replace standard CM (pig grower ration) at 0% (G_1_), 20% (G_2_), 30% (G_3_), and 40% (G_4_). The chayote meal consisted of the fruits and leaves of *S. edule* mixed in the ratio of 4:1. This total mixture was cooked for 30 min before feeding to the animals.

### Experimental procedure

After a provision of environmental and dietary adaptation period of 10 days, a 90-day feeding trial was conducted. During this period, the daily dry matter (DM) intake was recorded. The body weight of the animals was recorded before the start of the feeding trial and at 7 days interval during the experimental period. A digestion trial of 5 days was conducted by taking 3 animals from each group. Feeds offered and residues left were recorded daily during the digestion trial. Feces voided daily by the individual animal were weighed separately in previously weighed containers. The amount of feces collected at 24 h was quantified and representative samples were taken daily for aliquoting in the laboratory. Representative samples of each of the concentrate feed ingredients, chayote fruits, leaves, and meal used in the experiment, residual feeds, and fecal matter were analyzed in the laboratory for proximate principles as per the method described by AOAC [7], Fiber fractions as per the method described by Goering and Soest [8] and calcium as per the method described by Talapatra *et al*. [9].

### Statistical analysis

All the data were analyzed statistically using Statistical Packages for Social Sciences Software, Version 17.00 (SPSS Inc., Chicago, USA), and by the statistical method [10] for discussion and interpretation of results.

## Results and Discussion

### Chemical composition

Nutrient composition of chayote fruits, stems, leaves, and meal is presented in Table-1. It was observed that the DM (%) of boiled chayote meal, chayote fruit, chayote leaf, and chayote stem was 15.10, 5.92, 17.50, and 18.32, respectively. The chayote fruit contains less DM percentage (5.92) as compared to leaves and stem. Further, on DM basis, the chemical composition of cooked chayote meal, chayote fruit, chayote leaf, and chayote stem were 5.87, 5.97, 16.14, and 14.99%, respectively for total ash; 13.62%, 14.88%, 15.01%, and 14.58%, respectively for crude protein (CP); 0.70%, 0.83%, 1.15%, and 1.10%, respectively, for ether extract (EE); 11.73%, 7.53%, 12.10%, and 21.71%, respectively, for crude fiber (CF); and 55.53%, 70.79%, 55.60%, and 47.62%, respectively, for nitrogen-free extract (NFE). These observations were in agreement with Yoshimura [11] who reported that the chayote fruit contains 4.03% DM, and the DM contained 16.26% CP, 1.17% EE, 7.31% CF, 68.39% NFE, and 6.86% total ash, respectively. Similar observations were also observed by Nagarajaiah and Prakash [12] in *S. edule* dehydrated peels in which 100 g contained 15.15 g proteins, 45.24 g insoluble fiber, and 2.32 g EE.

**Table-1:** Nutrient composition of chayote (*S. edule*) fruit, stem, leaves, and meal.

Parameters	Chayote meal	Chayote fruit	Chayote leaf	Chayote stem
DM %	15.10	5.92	17.50	18.32
Total ash %	5.87	5.97	16.14	14.99
CP %	13.62	14.88	15.01	14.58
EE %	0.70	0.83	1.15	1.10
Crude Fiber %	11.73	7.53	12.10	21.71
NFE %	55.53	70.79	55.6	47.62
Acid insoluble ash %	0.02	0.01	0.05	0.03
Neutral detergent fiber %	38.02	29.34	33.86	41.67
Acid detergent fiber %	24.92	19.67	37.77	39.23
Calcium %	0.15	0.25	0.67	0.46
Phosphorus %	0.93	0.60	0.82	1.57

DM=Dry matter, NFE=Nitrogen free extract, CF=Crude fiber, EE=Ether Extract, CP=Crude protein, *S. edule=Sechium edule*

The ration for different experimental groups consisted of different levels of CM ingredients and chayote meal. The chemical composition of different experimental feed is presented in the Table-2. On laboratory analysis, the DM of diets offered to G_1_, G_2_, G_3_, and G_4_ was 86.63%, 86.35%, 87.21%, and 85.74%, respectively. The chemical composition of diets for G_1_, G_2_, G_3_, and G_4_ were 8.36%, 10.38%, 9.63%, and 9.56%, respectively, for total ash; 18.05%, 17.90%, 17.90%, and 18.14%, respectively, for CP; 0.67%, 1.74%, 1.77%, and 1.92%, respectively, for EE; 5.02%, 5.08%, 7.20%, and 7.41%, respectively, for CF; and 67.90%, 64.90%, 63.50%, and 62.97%, respectively, for NFE.

**Table-2:** Nutrient composition of experimental feed fed to different experimental groups.

Parameters	G_1_	G_2_	G_3_	G_4_
			
	Concentrate mixture	20%	30%	40%
DM %	86.63	86.35	87.21	85.74
Total Ash %	8.36	10.38	9.63	9.56
CP %	18.05	17.90	17.90	18.14
EE %	0.67	1.74	1.77	1.92
CF %	5.02	6.51	7.20	7.41
NFE %	67.9	64.9	63.5	62.97
Acid insoluble ash %	0.05	0.09	0.08	0.07
Neutral detergent fiber %	34.76	32.35	32.98	33.54
Acid detergent fiber %	10.32	13.95	17.35	19.51
Calcium %	0.27	0.27	0.28	0.24
Phosphorus %	1.01	0.79	0.82	0.84

DM=Dry matter, NFE=Nitrogen free extract, CF=Crude fiber, EE=Ether Extract, CP=Crude protein

### Feed intake

From the perusal of the Table-3, it is observed that the average DM intake (g/head/day) was 1263.27±212.18, 1115.97±153.87, 970.71±144.35, and 1055.69±113.83 for G_1_, G_2_, G_3_, and G_4_, respectively. The DM that the feed intake (kg/pig/day) was 1.33, 1.45, 1.36, and 1.25, respectively, for 0%, 5%, 10%, and 15% supplementation of cottonseed cake in the standard concentrate diet [13]. However, statistical analysis of the data revealed that the DM intake of feed by pigs of different groups did not differ significantly (p>0.05). The numerical reduction of DM intake in experimental groups may be attributed to more voluminous nature of the cooked chayote meal. The high moisture content of the experimental feed rendered it quite voluminous during feeding, which might have triggered the satiety center of the brain before the actual DM requirement of the animal was met [14], on incorporating ajar seed kernel in the diet of crossbred pigs, also observed the feed intake was 1.7 kg/day. Slight variations with the present observation may be due to breed differences as the animals used in the present study were small, non-descriptive type of pigs.

**Table-3:** Weekly DM intake (g/day) by pigs fed chayote meal.

Week	Attributes	G_1_	G_2_	G_3_	G_4_	Mean±SE	p
1	Concentrate mixture intake	601.06±94.32	435.44	335.95	282.84	477.22±50.98	0.19^NS^
	Cooked chayote meal	-	48.38	83.99	121.22		
	Total	601.06	483.82±46.55	419.93±38.18	404.05±24.90		
2	Concentrate mixture intake	942.93±222.50	646.35	421.54	392.53	687.19±116.61	0.17^NS^
	Cooked chayote meal	-	71.82	105.39	168.23		
	Total	942.93	718.17±116.40	526.93±66.29	560.76±61.24		
3	Concentrate mixture intake	983.42	842.61	562.99	629.50	880.67±149.45	0.67^NS^
	Cooked chayote meal	-	93.62	140.75	269.79		
	Total	983.42±222.04	936.24±159.95	703.74±106.55	899.29±109.28		
4	Concentrate mixture intake	978.09	878.44	659.58	753.95	963.92±187.40	0.92^NS^
	Cooked chayote meal	-	97.60	164.89	323.12		
	Total	978.09±236.42	976.04±195.39	824.47±170.02	1077.07±147.78		
5	Concentrate mixture intake	1173.56	867.01	771.69	732.75	1037.08±187.83	0.90^NS^
	Cooked chayote meal	-	96.33	192.92	314.04		
	Total	1173.56±248.38	963.35±219.13	964.61±154.77	1046.79±129.04		
6	Concentrate mixture intake	1170.45	973.26	793.62	729.35	1071.45±143.91	0.83^NS^
	Cooked chayote meal	-	108.14	198.40	312.58		
	Total	1170.45±213.21	1081.41±153.22	992.02±101.90	1041.92±107.31		
7	Concentrate mixture intake	1400.79	1100.09	982.52	820.88	1255.99±172.66	0.83^NS^
	Cooked chayote meal	-	122.23	245.63	351.81		
	Total	1400.79±237.44	1222.32±165.11	1228.16±152.85	1172.69±135.27		
8	Concentrate mixture intake	1432.99	1190.41	1051.03	891.15	1335.63±183.92	0.92^NS^
	Cooked chayote meal	-	132.27	262.76	381.92		
	Total	1432.99±231.34	1322.67±150.46	1313.79±200.46	1273.07±153.42		
9	Concentrate mixture intake	1467.58	1230.95	1009.68	893.56	1343.48±90.85	0.83^NS^
	Cooked chayote meal	-	136.77	252.42	382.96		
	Total	1467.58±239.21	1367.72±148.22	1262.10±226.86	1276.52±149.14		
10	Concentrate mixture intake	1459.79	1199.58	945.85	911.54	1319.29±183.51	0.76^NS^
	Cooked chayote meal	-	133.29	236.46	390.66		
	Total	1459.79±236.80	1332.86±151.74	1182.31±216.94	1302.20±128.64		
11	Concentrate mixture intake	1627.29	1386.68	961.40	866.70	1401.98±151.78	0.18^NS^
	Cooked chayote meal	-	154.08	240.35	371.44		
	Total	1627.29±240.32	1540.76±147.52	1201.74±124.18	1238.14±96.12		
12	Concentrate mixture intake	1580.85	1262.94	788.56	778.27	1270.41±169.83	0.21^NS^
	Cooked chayote meal	-	140.33	197.14	333.54		
	Total	1580.85±253.21	1403.27±142.01	985.70±162.97	1111.81±121.16		
13	Concentrate mixture intake	1603.80	1043.11	811.04	923.80	1274.08±139.74	0.33^NS^
	Cooked chayote meal	-	115.90	202.76	395.91		
	Total	1603.80±83.20	1159.01±204.60	1013.80±154.59	1319.71±116.56		

DM=Dry matter, NS=Non-significant, SE=Standard error

### Growth performance

**Figure-1:**
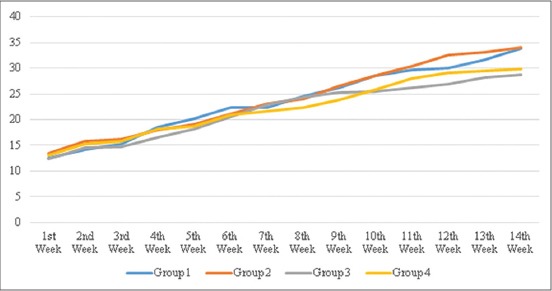
Weekly body weight changes (kg) of different treatment groups.

The body weight changes of the experimental pigs during the 14 weeks of experimental period has been depicted in Figure-1, and the performance of the local pigs fed on chayote meal as replacement of standard CM is presented in Table-4. It was observed that the average body weight gain was highest (21.22 ± 3.73 kg) in G_1_ and lowest in G_3_ (16.43±2.42 kg) for the experimental period of 90 days. The values for feed conversion ratio (FCR) observed during the experiment were 5.42±0.44, 4.93±0.17, 5.38±0.05, and 5.74±0.53 for G_1_, G_2_, G_3_, and G_4_, respectively. This was in agreement with Keoboualapheth and Mikled [15], who reported that 10%, 20%, and 30% of the rice bran replacement by *Stylosanthes guianensis* CIAT 184 foliage resulted in similar FCR. The close similarity may be attributed to the possibility of breed resemblance as an indigenous pig breed of Thailand was used in their study. Comparatively poor FCR was observed with the increasing replacement of CM with unconventional feedstuffs. In the present study, there was an increasing tendency of feed conversion efficiency values when higher amount of chayote meal was fed. This tendency was also observed by Halimani *et al*. [16] who fed Acacia leaf meals on growing Large White pigs. Pigs fed diets including leaf meals have been shown to have low nitrogen retention, which could explain the decline in weight gain [17]. The average daily gain (ADG) (kg) of the experimental animals in the present study was 0.24±0.04, 0.23±0.03, 0.18±0.02, and 0.18±0.02 for G_1_, G_2_, G_3_, and G_4_, respectively. Kumaresan *et al*. [18] observed that the ADG of nondescript local pigs of Mizoram in traditional backyard production system was 0.12 kg, which is parallel to the value observed during the experiment. It is evident that in most of the experiments the ADG of the animals fed standard CM was higher as compared to those that are fed with replacement of unconventional feedstuffs. In rural areas where availability of good quality feedstuffs is limited due to various problems, the condition can influence the quality and quantity of pig feed which subsequently influences the ADG of pigs [19, 20]. The values for ADG in animals fed chayote meal were comparable to the findings of Sotola [21] in which the ADG was 0.28 kg. The slight variation may be due to breed differences, feed composition, environmental factors, or other physiological parameters.

**Table-4:** Growth performance of pigs of different treatment groups.

Parameters	G_1_	G_2_	G_3_	G_4_	p
Initial body weight (kg)	12.61±2.34	13.42±1.96	12.37±1.77	13.09±2.12	0.98^NS^
Final body weight (kg)	33.83±5.62	34.00±4.23	28.80±5.12	29.83±5.34	0.79^NS^
Body weight gain (kg)	21.22±3.73	20.58±2.40	16.43±2.42	16.73±2.00	0.39^NS^
Total DM intake (kg)	114.96±20.55	101.55±13.30	88.34±14.22	96.07±8.69	0.34^NS^
FCR	5.42±0.44	4.93±0.17	5.38±0.05	5.74±0.53	0.87^NS^
ADG (kg)	0.24±0.04	0.23±0.03	0.18±0.02	0.18±0.02	0.42^NS^

ADG=Average daily gain, FCR=Feed conversion ratio, DM=Dry matter, NS=Non-significant

### Digestibility of nutrients

The digestibility coefficient of DM, CP, CF, and EE is presented in the Table-5. The values observed for DM digestibility (i.e. 78.62±1.91, 79.33±0.55, 77.25±3.89, and 76.31±0.85 for G_1_, G_2_, G_3_, and G_4_, respectively) were in agreement with Yadav *et al*. [22] who used sweet potato (*Ipomoea batatas*) vines for replacing the standard CM in pig and observed 64.93, 77.54, and 85.08% DM digestibility for 0%, 50%, and 100% replacement respectively. Similar observation was also found by Manh *et al*. [23] who reported that the DM digestibility was 87, 74, and 74 for 2%, 4%, and 6% replacement of CM by water hyacinth in the ration of pigs. The values for CP digestibility were in agreement with Manh *et al*. [23] who recorded the CP digestibilities as 79.0, 68.0, and 73.0 for 2%, 4%, and 6% levels, respectively. The results were also comparable to the observations of Madhava Rao *et al*. [24] in the study of inclusion of guava pomace at the level of 0%, 10%, 20%, and 30% (i.e. 76.5, 69.2, 70.3, and 68.9, respectively). The decrease in the digestibility of CP from G_2_ to G_4_ may be due to the higher CF content of the diets. Negative influence of dietary fiber on CP digestibility could be attributed to the lower availability of protein added with fiber source [25]. Low digestibility of protein may also be due to protein being bound by polyphenols and fiber or physically entrapped by fiber in the chayote meal when the leaves are included [26].

**Table-5:** Intake (g/head/day) and digestibility (mean±SE) of various nutrients in the four experimental groups.

Nutrients	G1	G2	G3	G4	p
DM					
Intake (g/head/day)	1320.45	1538.33	1323.83	1311.16	0.77^NS^
Digestibility (mean±SE)	78.62±1.91	79.33±0.55	77.25±3.89	76.31±0.85	
CP					
Intake (g/head/day)	247.62	266.66	240.38	239.41	0.97^NS^
Digestibility (mean±SE)	79.31±3.35	77.28±4.20	77.89±3.94	77.30±1.49	
CF					
Intake (g/head/day)	70.52	75.16	96.91	96.99	
Digestibility (mean±SE)	50.92±3.79	49.99±2.54	52.97±7.14	60.97±1.02	0.14^NS^
EE					
Intake (g/head/day)	6.03	25.14	23.44	25.48	
Digestibility (mean±SE)	51.38^a^±2.56	76.45^b^±1.69	76.03^b^±3.94	75.79^b^±1.22	0.00**

Values with similar superscripts (row-wise a, b) did not differ significantly (p>0.01), EE=Ether extract, CF=Crude fiber, CP=Crude protein, DM=Dry matter, SE=Standard error

The figures observed in CF digestibility were in agreement with the value (54.5) which was reported by Totsuka *et al*. [27] in growing-finishing pigs by feeding cassava as a replacement of standard CM. Fibrous components of the diet are poorly digested in the small intestine of the pig and provide substrates for microbial fermentation in the large intestine. On replacing standard CM with 0%, 3%, 6%, 9%, and 12% of fermented cassava pulp, and reported the EE digestibility of was found to be 67.0%, 68.1%, 79.8%, 77.4%, and 81.8%, respectively, which was reported by Huu and Khammeng [28]. Perondi *et al*. [29] also reported crude fat digestibility coefficient of 83.23% when growing-finishing pigs were fed with 16% replacement of standard CM with passion fruit seed meal. These observations agree with the EE digestibility observed in the current experiment. Noblet and Perez [30] reported that the amount of digestible EE content was linearly and positively related to the dietary EE content and negatively affected by the dietary NDF content. The decreasing trend of EE digestibility in the present study may be due to the CF contents in the experimental feed.

The cost of feeding the experimental animals was calculated taking into consideration the minimal cost of the chayote fruits at wholesale price in Mizoram. The cost per kg body weight gain of experimental pigs in G_1_, G_2_, G_3_ and G_4_ was Rs. 89.15, Rs 78.77, Rs 82.64, and Rs 84.89, respectively. In Mizoram, chayote is grown mostly in the family plots or the vegetable garden. If it is available at farmer’s garden, it needs not to be purchased from the market. Accordingly, the cost per kg weight gain may be further reduced and it was calculated to be Rs 89.15, Rs 73.02, Rs 70.66, and Rs 66.02 for G_1_, G_2_, G_3_, and G_4_, respectively.

## Conclusion

It can be concluded that feeding of chayote meal at different levels by replacing standard grower ration does not have any adverse effect on the growth performance and nutrient utilization of the pig. Therefore, in the pig’s diet, standard grower ration may be replaced up to 40% by chayote meal safely for better economic returns by the farmers. The same experimental feed may also be tested in large exotic breeds of pigs for further scientific information.

## Authors’ Contributions

JL carried out the experiment and drafted the manuscript. AKS designed the experiment, guided during the experiment and helped in the analysis of the data. Both authors read and approved the final manuscript.
